# Diffusion of Technology in the Teaching of Neuroanatomy in Times of Pandemic: A Medical and Academic Perspective on Learning

**DOI:** 10.3389/fsurg.2022.888546

**Published:** 2022-07-04

**Authors:** Herika Karla Negri Brito, Ana Cristina Veiga Silva, Luís Felipe Gonçalves de Lima, Joaquim Fechine de Alencar Neto, Otávio da Cunha Ferreira Neto, Nilson Batista Lemos, Artêmio José Araruna Dias, Andrey Maia Silva Diniz, Luana Moury Fernandes Sanchez, Melissa Helena Rodrigues Silva, Luís Bandeira Alves Neto, Arthur Oliveira Lira, Luís Felipe Ferreira Marques, Maria Luísa Rocha, Luiz Severo Bem Junior, Marcelo Moraes Valença, Hildo Rocha Cirne de Azevedo Filho, Débora Maria Brito de Pinho

**Affiliations:** ^1^ Department of Neurosurgery, Hospital da Restauração, Recife-PE, Brazil; ^2^ College of Medical Sciences, Unifacisa University Center, Campina Grande-PB, Brazil; ^3^ Medicine department, Catholic University of Pernambuco, Recife-PE, Brazil; ^4^ Medicine department, Federal University of Paraíba, João Pessoa-PB, Brazil; ^5^ Medicine department, University Maurício de Nassau, Recife-PE, Brazil; ^6^ Medicine department, University of Pernambuco, Recife-PE, Brazil; ^7^ College of Medical Sciences, Mato Grosso State University, Cácere-MT, Brazil; ^8^ Brasília University Center, Brasília-DF, Brazil; ^9^ Neuroscience Post-Graduate Program, Federal University of Pernambuco, Recife, Brazil

**Keywords:** COVID-19, teaching-learning, neuroanatomy, technology, virtual reality

## Abstract

The Covid-19 pandemic has caused major changes in many sectors of society worldwide. The issue of medical education stands out since it had to adapt to the rules of social isolation, ensuing discussions about the computerization of teaching methodology, particularly in neuroanatomy. In particular, the latter showed satisfactory adaptability to new technologies and highly promising learning results. During this review, we aim to evaluate the current state of neuroanatomy teaching and evaluate the possibilities of incorporating technology into teaching–learning of human anatomy in a post-pandemic world.

## Introduction

On March 11, 2020, the World Health Organization (WHO) (1) formally declared Covid-19 a pandemic. Health systems, the economy, and international education systems have been and still are seriously affected, requiring measures in health, public distancing policies, and quarantines.

Educational institutions, at all levels, both schools and Universities, were closed in 186 countries worldwide, indefinitely, impacting more than 91% of the world's student population (2). Since medical education is conducted face-to-face, it is worrisome in several respects since it is based on a historical pedagogical foundation.

Throughout history, some disciplines, such as Human Anatomy, have had more conservative teaching methods. Frederick II (1194–1250), for example, issued a decree in 1,231 which stated that a human body had to be dissected every five years for anatomical studies and those mandatory dissections were required for anyone studying medicine (3). In the Renaissance period, scholars like Leonardo Da Vinci also used dissection to deepen their knowledge of human anatomy.

Over the years, however, the millenary pragmatism of using corpses in the teaching of human anatomy has slowly been weakened. The study of anatomy has been enhanced with the use of three-dimensional applications with virtual reality in many universities around the globe to supplement medical education, which was previously limited to cadavers (4).

Due to social distancing and a poor understanding of biological risks related to cadavers during the pandemic, teaching of human anatomy was faced with many difficulties and uncertainties. Nevertheless, it has provided an opportunity for cadaver-free teaching to be re-invented and improved. As a result, the pandemic caused by the new Coronavirus has the potential to catalyze a revolution in the teaching of human anatomy, especially in the study of the nervous system, that under normal circumstances would probably take a considerable amount of time to happen.

In this review, we will evaluate the current state of neuroanatomy teaching in the world and, in addition, discuss ways to improve the use of technology in teaching–learning of human anatomy in a post-pandemic era.

## Methods

The purpose of this review is to explore the literature on Medicine and Teaching in articles found in databases like PubMed and Virtual Health Library (VHL). In addition, other articles and editorials were selected manually via Google Scholar. The search for articles on databases obeyed the following keywords: Covid-19, 2019-nCOV, SARS-CoV-2, Medical Students, Neuroanatomy, Anatomy, Virtual Reality, Technology. Boolean operators “OR” and “AND” were accurately applied to optimize search. As an initial inclusion criterion, after applying the Boolean operator “OR” and “AND”, the articles should have at least two keywords included in the title or abstract. The search was restricted to articles written in Portuguese, English or Spanish. There was no temporal restriction regarding the date of publication of the article and its eligibility for the study. After the application of the inclusion methods already mentioned, abstracts and the articles were read. Only those considered to be substantial for promoting the discussion and those which achieved the goals of this study were selected and included, resulting in 25 selected articles.

## Results

From the studies of neuroanatomy and the perception of the complexity inherent to the understanding of neural morphology of the intricate nervous system, the need for tools and methods that provide visualization of the structures in full becomes evident. Based on the analysis of the selected articles, we can see how medical schools face challenges in teaching this content because of technical limitations, most notably the low conservation level associated with cadaveric pieces and the difficulty inherent to identifying structural elements in situ.

In the twentieth century, with the advent of the Internet, several innovative teaching mechanisms have become available. These mechanisms range from 3D visualization of pieces to immersion in virtual worlds, yet these tools are still restricted in medical education. Students are limited to studying cadavers in class, which limits their resources when it comes to learning. However, the Sars-Cov-2 pandemic, in the year 2020, was responsible for an abysmal change in the way anatomy classes were taught. To compensate for the reduction of student contact with anatomical parts in laboratories, medical universities have adopted a number of technological mechanisms. These mechanisms are described in the selected articles.

Although cadavers are necessary for the study of anatomy, particularly the nervous system, new teaching techniques are essential for a holistic and comprehensive understanding of organs and systems. Furthermore, by integrating theory and technology, we can effectively transfer information, since students can visualize the neuroanatomy in a three-dimensional way, understanding in a more critical way the development of the structures, and hence enhancing medical education. Finally, the pandemic, as a driving factor for the adoption of mechanisms, as described in the selected articles, was the main aspect responsible for the dissemination of these tools, a reality that in the future will bring benefits and advances of great importance in treatment techniques of patients and intervention on their diseases, especially in cases of surgical planning (5).

## Discussion

### Tradicional Education of Human Anatomy

From the beginning of anatomy studies, the use of cadavers and organs constitutes a standardized method of transmitting knowledge from the teacher to the students to build technical-scientific knowledge of the studied parts. However, even though cadavers are the best means of teaching anatomy to medical and health sciences students, there are a great ethical, financial, and supervisory restrictions for their use (6). Moreover, although anatomy is still one of the main areas of medical education, universities have decreased the hours allocated to anatomy teaching in favor of applied clinical work. The launch of virtual and augmented reality devices allows learning to take place through practical immersive experiences making teaching–learning more conceptualized and, consequently, transforming future health professionals more prepared for the new times of professional performance (7).

**Figure 1:**
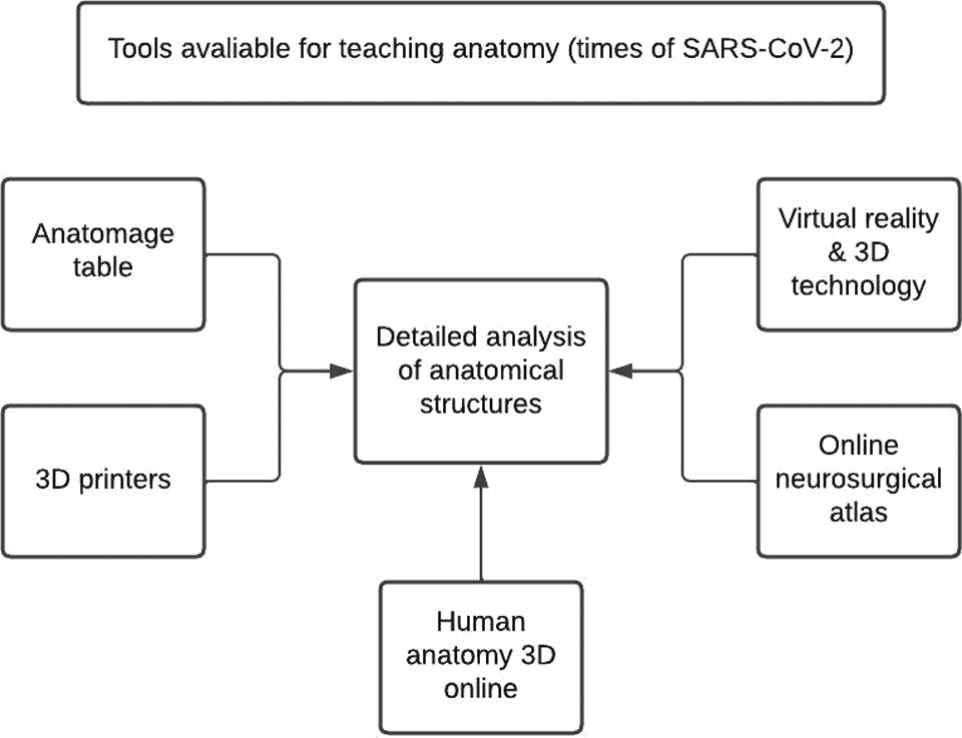
Tools adopted for greater learning in the teaching of anatomy and better visualization of structures, in Covid-19 times.

Neuroanatomy teaching becomes more powerful when combined with certain Technologies, as described in Figure 1. Because the nervous system, whether central or peripheral, is composed of a variety of complex structures characterized by sulcus, gyrus, and neurovascular structures, understanding their morphology facilitates the understanding of the functional areas and the diagnosis of their topography.

### Impositions of the Pandemic in the Medical Teaching of Neuroanatomy

As a result of the pandemic, methods that allied technology to teaching that stimulate active learning can be developed in place of passive teaching when students receive knowledge for classes through lectures without proactivity and, therefore, with reduced retention of knowledge (8).

Systematically adopting realistic simulations of neuroanatomical visualization tools, illustrative videos, and applications accessible directly to students, as well as printed 3D models that can be handled and studied by every student, among other general strategies, such as e-books and recorded video classes, are used for good anatomy learning. These tools will be fundamental in the future development of the profession, since, in addition to the analysis of imaging tests, a situation that is part of the routine of many physicians, the use of these mechanisms can be the differential at the time of planning as detailed by Landi et al. (9), and execution of medical interventions, especially in the surgical field (9).

Besides the difficulties of handling cadavers and human organs in the teaching of so-called modern neuroanatomy, the technological environment in the field of teaching makes teaching easier. Virtual reality is a technology that is spreading in several areas of our society, from the entertainment sector to the industrial sector. There are many advantages in education because it is possible to take advantage of almost any object or go anywhere in a unique way (10).

Another aspect that the Covid-19 pandemic favors the strengthening of new neuroanatomy teaching technologies is the biological risk of contamination by students and their teachers when they are in contact with biological substances, for this reason, the number of cadavers received by universities has drastically reduced (11).

The body that is given may be infected with the new coronavirus, and it is not feasible to test the corpse, as the screening kits are insufficient even for all suspected living cases. Furthermore, negative results may not exclude the actual Covid-19 infection (11). In addition, Covid-19 also warned of the need to use personal protective equipment (PPE) by all, in view of the risk of contamination of other agents that may be in the cadaver, despite the conservation and cleaning formulations.

Technology in medical education overcomes barriers by combining traditional theory with practical application facilitated by certain equipment and systems. Teaching and evaluation of the students’ procedural skills, using virtual reality, presented satisfactory results (12). Therefore, contemporary technologies mark the scenario of medical education, bringing more knowledge and practices to students and, also, practicality in the exchange of information between teachers.

### 3D Printers

One of the technologies in consolidation in the era of computerization of medical education is the use of printers in 3 dimensions, since it produces low-cost pieces but with high reproducibility of the anatomical characteristics of real cadaveric parts (13). These printing machines are an important tool to complement the study in real cadavers, for in many universities there is an important deficit in this aspect, due to scarcity of bodies and a high degree of damage of existing ones, making it difficult for students to perform detailed analyses and in small study groups. This problem is even more critical and worrisome in the discipline of neuroanatomy since the structures are more sensitive to time and wear from use in handling, their small size and fragility of dissection.

An important advantage of using these machines is that each student can handle, study in the laboratory or even at home with more time in contact with the piece. Thus, you can make comparations with atlases, make hypotheses and raise questions to the teacher, further stimulating the development of research and active teaching skills (13).

### Anatomage

Anatomage Table is a fully segmented 3d human anatomy application. The Anatomage Table is a system that promotes a deeper study of anatomy and is therefore being adopted by many medical schools worldwide. Its technology is advanced and combines several other – X-ray, ultrasound, CT scan, magnetic resonance – in order to build images.

In the literature, there are several studies with different applications for Anatomage. The latter was reported in a study with clinical application and surgical planning in oral and maxillofacial surgery (14). There are studies involving the teaching of radiology using Anatomage and its results were positive (15). However, most of the studies found involved the teaching of anatomy and neuroanatomy, using the Anatomage tool associated or not, with the use of cadavers, resulted in good performance of the students and perceived enthusiasm (16, 17).

### Other Technologies Associated With the Teaching of Neuroanatomy

There are technological tools that allow the visualization and anatomical study of imaging such as magnetic resonance imaging (MRI) and computed tomography (CT). This alternative study applied since the early years of graduation, being inserted in the discipline of Neuroanatomy, allows the student a greater understanding of anatomical spatial relationships, increasing the efficiency of study and interest in anatomy, as well as greater motivation in learning (18).

The use of images of medical exams in the teaching of anatomy provides in vivo visualization of the anatomical structure and physiology, as well as information about pathological processes, allowing the academic to learn more effectively about the anatomy and understanding of the analysis of these exams, which will be fundamental in their professional performance (19).

Another important tool in the process of diffusion of neuroanatomy study is 3D visualization technology. Although it is still an instrument of high acquisition cost by universities, it has important advantages in the learning process since spatial visualization from different angles allows greater understanding of the complex neural structures. Besides that, students assume a role of proactivity in the face of the study, being able to investigate and analyze in detail the neuroanatomy, use the zoom tool, rotate the model under an axis, touch the structures and obtain more information about it (20).

Virtual reality, in association with 3D technology, for the study of neuroanatomy is a good way to study not only for academics but also for residents and even for surgeon’s surgical planning (21).

In the study conducted by Stepan et al. (22). It was concluded that neuroanatomical study through virtual reality was as effective as traditional teaching methods, having been proven by testing research groups. In addition, virtual reality technology proved to be more advantageous in terms of stimulating teamwork, engaging in activity and motivation for learning (22).

Previous studies, such as that of Rhem, 1995 (23) showed that active teaching increases the understanding and retention of knowledge in a higher way than traditional methods. Through the use of technology in medical education it will be possible to contemplate all medical students, each cycle with its own solutions and adapted to its peculiarities (20).

### Neuroanatomy Online

Although teaching anatomy and neuroanatomy requires classically learning and face-to-face monitoring, the manipulation of corpses and anatomical dissection workshops have caused insecurity in students and teachers during the pandemic. However, this discipline, due to applications of technological tools, shows a greater capacity to adapt to the new times including promising chances of achieving quality and efficiency in distance learning (24).

As evidence of the growth of this trend, “The Neurosurgical Atlas,” a free atlas available on the internet obtained a high demand from new users, especially in countries whose effects of the pandemic were more remarkable (24).

At the University of Cambridge, the Atlas of Human Anatomy 3D Visible Body was adopted for medical students, as well as tutorials created using VH Dissector Touch Software (Touch of Life Technologies, Inc.) as the dissection of cadavers was interrupted. This made it possible for the transition to online education to be more comfortable for students. In addition, theoretical classes were given on history of anatomical dissection, the process of donation of bodies and methods of conservation of cadavers through the Zoom platform (25).

At Oxford University, a structure is being studied and planned with various anatomy teaching instruments, including “Instant Anatomy” and “Acland's Anatomy,” as well as annotations, diagrams and videos (25).

At the University of Edinburgh, anatomy teaching became remote. Resources and support have been put in place for students to obtain online access training using platforms such as VLE (LEARN), Microsoft Teams, and Kaltura Capture (25).

The results of teaching online neuroanatomy are not fully elucidated yet, if this method leaves gaps in knowledge, due to its limitations, such as the impossibility of handling cadaveric parts and dissection. This aspect will need to be analyzed and discussed in the coming months and years, along with the long-term impact on student knowledge and professional skills (25).

Therefore, it is the duty of medical education institutions, regarding the pandemic, to reinvent themselves to apply computer tools to the process of training new students. Of course, as long as an extensive analysis is performed on how these methods can be implemented, so as to ensure efficiency and solidity of study. Teachers and students will be able to overcome the new reality and, with this, the obstacles that once existed will therefore be knocked down and the doors to a new future will be open (8).

### Learning Methods and Teaching Integration

In view of all these limitations imposed by the pandemic scenario, despite the introduction of new tools for the study of neuroanatomy, the effectiveness and quality of the study are fundamental mechanisms for better learning and absorption of the content, especially with the deficits, resulting from social isolation in conventional teaching methodology. In this scenario, three possible mechanisms to enhance learning, in conjunction with the digital and online teaching mechanisms described in the article, can be applied, such as the testing effect, the active recovery and the spaced repetition (26). Regarding the testing effect, there is an analysis that the consolidation of absorbed information becomes more effective and there is a higher retention of data after the student tests himself, through quizzes for example, on the studied subject (27).

Furthermore, Augustin, M. (26) portrays that the effort to remember the studied subjects is more effective than a passive method of study. Spaced repetition, initially developed by Ebbinghaus, despite presenting limitations in the studies described at the time, for not having a test and control group in its analysis and using only isolated words for memorization, has been widely disseminated today. Methods such as the use of flashcards (28) and applications of study by repetition, such as Anki, are essential for the effective application of this study mechanism. All these learning mechanisms, combined with digital implementations and advances in the field of teaching, are possible means to achieve a wider transmission and absorption of neuranatomical knowledge reducing the serious consequences of the pandemic in this respect.

Through analysis and reflection of the current pandemic of Covid-19, it is observed that, undoubtedly, technology has gained notoriety in medical education, especially in the discipline of Human Neuroanatomy. Our review shows that Neuroanatomy is a more promising path and capable of having digital technologies allied to traditional cadaveric study, due to the challenges of this discipline which accompanies students during medical graduation.

Finally, it is clear that the union of technological and innovative tools and effective study techniques are paramount for the teaching of neuroanatomy to become more complete during periods of limited social contact. Thus, further investigations with students, teachers and educational specialists should be done in order to verify better ways to carry out neuroanatomical studies through complementary technologies, in addition to analyzing, in different contexts, their real efficacy and applicability.

## Limitations of the Study

This study is a literature review. However, to date, a limited number of studies on the diffusion, evaluation and financial viability of the use of technology in the teaching of neuroanatomy in times of pandemic and today have been published. Therefore, the results obtained are a reflection of the previously published literature and are not a model study for its wide use in the academic environment. In addition, the low number of publications and evaluations on the topic can generate distorted results, limiting the generalization of our conclusions.

## Conclusion

In summary, the diffusion of technology in the teaching of neuroanatomy in times of pandemic is an important topic that needs to be discussed within the scope of medical training, since teaching based on observation and dissection of cadavers requires care in handling, due to deterioration inherent to the learning process, the need for continuous acquisition/conservation of cadavers and the difficulty of visualizing delicate structures that are not easily accessible by dissection. Therefore, the use of 3D images, anatomage and virtual reality emerges as an alternative and tool to add to the learning of neuroanatomy, as it is believed, as published studies point out, that their use is an effective option for the consolidation of learning in neuroanatomy, especially in cases where the acquisition of cadavers is not very feasible and expensive for the educational institution.

## Author Contributions

Conceptualization: HKN, ACV, LFG. Methodology: JFA, OCF, NBL, AJA, AMS, LMF. Research: MHR, LBA, AOL, LFF, MLR. Supervision: HKN, LSB, HRC, DMB. Project management: JFA, OCF, NBL, AJA, AMS, LMF. Resources: LFG, LSB, MMV, HRC, DMB. Data curation: JFA, OCF, NBL, AJA, AMS, AOL, LBA. Software: JFA, OCF, NBL, AJA, AMS, LMF. Visualization: MHR, LBA, AOL, LFF, MLR. Writing: JFA, OCF, NBL, AJA, AMS, LMF, LFG. Review and Editing: HKN, HRC, LSB, DMB, MMV, LFG. All authors contributed to the article and approved the submitted version.

## References

[1] World Health Organization. https://www.who.int/director-general/speeches/detail/who-director-general-sopening-remarks-at-the-media-briefing-on-covid-19-11-march-2020.

[2] United Nations Educational, Scientific and Cultural Organization. Education: From disruption to recovery. UNESCO. (2020). Retrieved from: https://en.unesco.org/covid19/educationresponse#durationschoolclosures

[3] Ghosh SK. Human cadaveric dissection: a historical account from ancient Greece to the modern era. Anat Cell Biol. (2015) 48(3):153–69. 10.5115/acb.2015.48.3.153 26417475 PMC4582158

[4] Keenan ID ben Awadh A. Integrating 3D visualisation technologies in undergraduate anatomy education. Adv Exp Med Biol. (2019) 1120:39–53. 10.1007/978-3-030-06070-1_4 30919293

[5] Hennessy CM Smith CF. Digital and social media in anatomy education. Adv Exp Med Biol. (2020) 1260:109–22. 10.1007/978-3-030-47483-6_6 33211309

[6] Moro C Štromberga Z Raikos A Stirling A. The effectiveness of virtual and augmented reality in health sciences and medical anatomy. Anat Sci Educ. (2017) 10(6):549–59. 10.1002/ase.1696 28419750

[7] Border S. Assessing the role of screencasting and video use in anatomy education. Adv Exp Med Biol. (2019) 1171:1–13. 10.1007/978-3-030-24281-7_1 31823235

[8] Chen CH Mullen AJ. COVID-19 can catalyze the modernization of medical education. JMIR Med Educ. (2020) 6(1):e19725. URL: https://mededu.jmir.org/2020/1/e19725. 10.2196/19725 32501809 PMC7294998

[9] Landi F O'Higgins P. Applying geometric morphometrics to digital reconstruction and anatomical investigation. Adv Exp Med Biol. (2019) 1171:55–71. 10.1007/978-3-030-24281-7_6 31823240

[10] Izard SG Juanes Méndez JA Palomera PR. Virtual reality educational tool for human anatomy. J Med Syst. (2017) 41(5):76. 10.1007/s10916-017-0723-6 28326490

[11] Singal A Bansal A Chaudhary P. Cadaverless anatomy: darkness in the times of pandemic Covid-19 [published online ahead of print, 2020 May 28]. Morphologie (2020) 104(346):147–50. 10.1016/j.morpho.2020.05.003 32518047 PMC7254017

[12] Kaufman DM Bell W. Teaching and assessing clinical skills using virtual reality. Stud Health Technol Inform. (1997) 39:467–72. 10.3233/978-1-60750-883-0-467 10168941

[13] Wen CL. Homem virtual (ser humano virtual 3D): a integração da computação gráfica, impressão 3D e realidade virtual para aprendizado de anatomia, fisiologia e fisiopatologia. Grad. (2016) 1(1):7–15. 10.11606/issn.2525-376X.v1i1p7-15

[14] Brucoli M Boccafoschi F Boffano P Broccardo E Benech A. The Anatomage Table and the placement of titanium mesh for the management of orbital floor fractures. Oral Surg Oral Med Oral Pathol Oral Radiol. (2018) 126(4):317–21. 10.1016/j.oooo.2018.04.006 29784601

[15] Custer T Michael K. The utilization of the anatomage virtual dissection table in the education of imaging science students. J Tomogr Simul. (2015) 1(1):1–5. 10.4172/jts.1000102

[16] Brown J Stonelake S Anderson W Abdulla M Toms C Farfus A Medical student perception of anatomage – a 3D interactive anatomy dissection table. Int JSurg. (2015) 23(1):17–8. Retrieved from: 10.1016/j.ijsu.2015.07.053

[17] Fyfe G Fyfe S Dye D Crabb H. Use of Anatomage tables in a large first year core unit. In: Carter H Gosper M Hedberg J, editors. Proceedings of the Electric Dreams 30th Ascilite Conference. Sydney, NSW: Macquarie University (2013). p. 298–302.

[18] Estai M Bunt S. Best teaching practices in anatomy education: a critical review. Ann Anat. (2016) 208:151–7. 10.1016/j.aanat.2016.02.010 26996541

[19] de Barros N Rodrigues CJ Rodrigues AJ Jr de Negri Germano MA Cerri GG. The value of teaching sectional anatomy to improve CT scan interpretation. Clin Anat. (2001) 14(1):36–41. 10.1002/1098-2353(200101)14:1<36::AID-CA1006>3.0.CO;2-G 11135396

[20] Allen LK Eagleson R de Ribaupierre S. Evaluation of an online three-dimensional interactive resource for undergraduate neuroanatomy education. Anat Sci Educ. (2016) 9(5):431–9. 10.1002/ase.1604 26990135

[21] Jones DG. Three-dimensional printing in anatomy education: assessing potential ethical dimensions. Anat Sci Educ. (2019) 12(4):435–43. 10.1002/ase.1851 30554454

[22] Stepan K Zeiger J Hanchuk S Del Signore A Shrivastava R Govindaraj S. Immersive virtual reality as a teaching tool for neuroanatomy. Int Forum Allergy Rhinol. (2017) 7(10):1006–13. 10.1002/alr.21986 28719062

[23] Rhem J. Close-up: going deep. Natl Teach Learn Forum. (1995) 5(1):4. 10.1002/ntlf.30049

[24] Aikaterini D Marinos GS John GH Deepa J Panagiotis D Michail S. Medical and surgical education challenges and innovations in the COVID-19 Era: a systematic review. In Vivo. (2020) 34(3 Suppl):1603–11. 10.21873/invivo.11950 32503818 PMC8378024

[25] Brassett C Cosker T Davies DC Dockery P Gillingwater TH Lee TC COVID-19 and anatomy: stimulus and initial response. J. Anat. (2020) 00:1–11. 10.1111/joa.13274 PMC7361297 32628795

[26] Augustin M. How to learn effectively in medical school: test yourself, learn actively, and repeat in intervals. Yale J Biol Med. (2014) 87(2):207–12. https://www.ncbi.nlm.nih.gov/pmc/articles/PMC4031794/24910566 PMC4031794

[27] Roediger HL Karpicke JD. Test-enhanced learning: taking memory tests improves long-term retention. Psychol Sci. (2006) 17(3):249–55. 10.1111/j.1467-9280.2006.01693.x 16507066

[28] Schmidmaier R Ebersbach R Schiller M Hege I Holzer M Fischer MR. Using electronic flashcards to promote learning in medical students: retesting versus restudying. Med Educ. (2011) 45(11):1101–10. 10.1111/j.1365-2923.2011.04043.x 21988625

